# Seed Wings Optimize the Regulation of Temperature and Light on Smith Fir Seed Germination Timing

**DOI:** 10.3390/plants15030508

**Published:** 2026-02-06

**Authors:** Yanyan Li, Ziling Yang, Qian Yan, Guoyan Wang, Songlin Shi, Jingji Li, Peihao Peng

**Affiliations:** 1 State Key Laboratory of Geohazard Prevention and Geoenvironment Protection, Chengdu University of Technology, Chengdu 610059, China; lyy1503640@163.com (Y.L.); shisonglin17@cdut.edu.cn (S.S.); lijingji2014@cdut.edu.cn (J.L.); 2 College of Geography and Planning, Chengdu University of Technology, Chengdu 610059, China; 15086852160@163.com; 3 School of Ecology and Environment, Tibet University, Lhasa 850000, China; yangzl19960418@163.com; 4 Institute of Ecological Resources and Landscape, Chengdu University of Technology, Chengdu 610059, China; peihaop@163.com

**Keywords:** *Abies georgei* var. *smithii*, environmental adaptation, gymnosperm, germination strategy, light sensitivity, seed wings

## Abstract

Seed wings are widely recognized for facilitating dispersal and influencing germination in angiosperms, but their functional role in gymnosperm germination is poorly understood. To assess the effect of seed wings on the germination ecology of Smith fir (*Abies georgei* var. *smithii*), we evaluated the germination of three seed treatments—intact seeds, mixed seeds (de-winged seeds mixed with detached wings), and de-winged seeds—under varying light and temperature conditions. Results showed that de-winged seeds achieved a final germination percentage of 48.5 ± 5.0%, which was significantly higher than that of intact seeds (26.0 ± 2.4%) and mixed seeds (32.5 ± 3.5%) (*p* < 0.001), confirming that seed wings significantly inhibit germination. There was no significant difference between intact and mixed seeds, and both were significantly lower than de-winged seeds (*p* < 0.001), suggesting that germination inhibition is likely mediated by chemical inhibitors in the wings rather than mechanical restriction. Optimal germination occurred at 15/2 °C–25/5 °C, while light significantly enhanced germination under cold conditions (5/1 °C), indicating conditional dormancy. These findings suggest that seed wings optimize the regulation of germination timing by imposing chemical inhibition that, combined with conditional dormancy, delays germination until favorable spring conditions, reflecting an adaptive strategy to seasonal environmental cues in subalpine ecosystems.

## 1. Introduction

Seed dispersal and germination are fundamental processes in plant life cycles, shaping species distributions, community structures, and ecosystem functions [1,2]. Dispersal reduces competition by enabling seeds to escape parental influence and colonize new habitats, enhancing seedling establishment. Germination, the transition from dormancy to active growth, is crucial for optimizing seedling survival [3]. These interconnected processes govern plant establishment, linking reproductive success to offspring survival [4]. Understanding their roles and interactions across ecological contexts is vital for evaluating plant adaptation and population persistence in changing environments.

Environmental cues, particularly temperature and light, serve as critical signals regulating seed germination and determining seedling survival in natural ecosystems [5]. To adapt to these fluctuating environmental factors, plants have evolved seed dormancy as a key bet-hedging strategy. Among its various forms, physiological dormancy is the most common, including conditional dormancy, where seeds cyclically transition between dormant and non-dormant states depending on external conditions [6]. This flexibility allows seeds to delay germination under unfavorable conditions and synchronize emergence with periods that maximize seedling establishment, particularly in ecosystems characterized by strong seasonality, such as temperate and alpine regions [7,8].

Beyond physiological mechanisms, physical structures like the seed coat, wings, and other appendages also play a role in germination regulation. Although dormancy and dispersal have traditionally been examined as distinct processes, growing evidence suggests that dispersal-related structures can directly influence germination timing and success [9,10]. A large group of plants possess seeds with additional appendages, such as winged perianth or bracteole, which significantly influence seed germination in many angiosperms. A recent study on *Acer tegmentosum* found that the pericarp acts as a “thermal filter” that specifically inhibits germination under high-temperature conditions—possibly by restricting oxygen uptake—whereas removing the pericarp allows seeds to germinate at the same temperatures [11]. This mechanism prevents premature seedling growth during high summer temperatures. This regulatory role of seed appendages on germination is also observed in other taxa. Bracteoles in *Atriplex* species, pappi in *Taraxacum*, and seed wings in *Ulmus* and *Salsola* species can inhibit germination by creating mechanical barriers [12], inducing light requirements [13], releasing chemical inhibitors like abscisic acid [14,15], or restricting water absorption and light availability [3,16]. These findings highlight that dispersal structures are not merely passive agents of dissemination but are functionally linked to germination regulation.

While the ecological functions of seed appendages are well documented in angiosperms, comparable structures also occur in many gymnosperms, yet their functional roles remain poorly understood. Many gymnosperms, particularly in the *Pinaceae* and *Cupressaceae*, also possess seed wings, as observed in *Picea purpurea*, *Abies forrestii*, *Pinus bungeana*, *Pinus massoniana*, and *Larix lyallii*. These gymnosperm-dominated forests, which are vital components of boreal taiga, temperate and subtropical subalpine ecosystems, cover over 40% of the global forested area and are crucial for carbon sequestration and ecological stability [17,18,19]. However, it remains unclear whether seed wings in gymnosperms, as in angiosperms, play a regulatory role in germination, which hinders understanding of their ecological adaptations and responses to global change.

This study focuses on the seeds of the Smith fir, a dominant tree species at the alpine treeline across the eastern Tibetan Plateau, where it faces harsh low temperatures and a short growing season. Previous studies show that Smith fir seeds germinate optimally between 15 °C and 20 °C, suggesting that germination could theoretically occur in spring, summer, or autumn under suitable climatic conditions. The seeds possess membranous wings and are typically dispersed in October, but no substantial germination has been observed during the autumn or winter in natural settings [20]. This raises the key ecological question: how do Smith fir seeds avoid germinating in autumn and instead delay germination until the following spring?

The cause of delayed germination in Smith fir (*Abies georgei* var. *smithii*) seeds, whether due to dormancy or inhibition by seed wings, remains unclear. We hypothesize that Smith fir seeds delay germination through dormancy and wing-mediated regulation, aligning germination with seasonal environmental conditions after spring snowmelt in subalpine regions. To address these gaps, we propose the following research questions: (1) Do Smith fir seeds exhibit dormancy, and how do they respond to seasonal environmental conditions in the field? (2) In addition to dispersal, do seed wings influence germination timing or success, and if so, by what mechanisms?

## 2. Results

### 2.1. Measurement of Seed Morphological Characteristics

**Figure 1:**
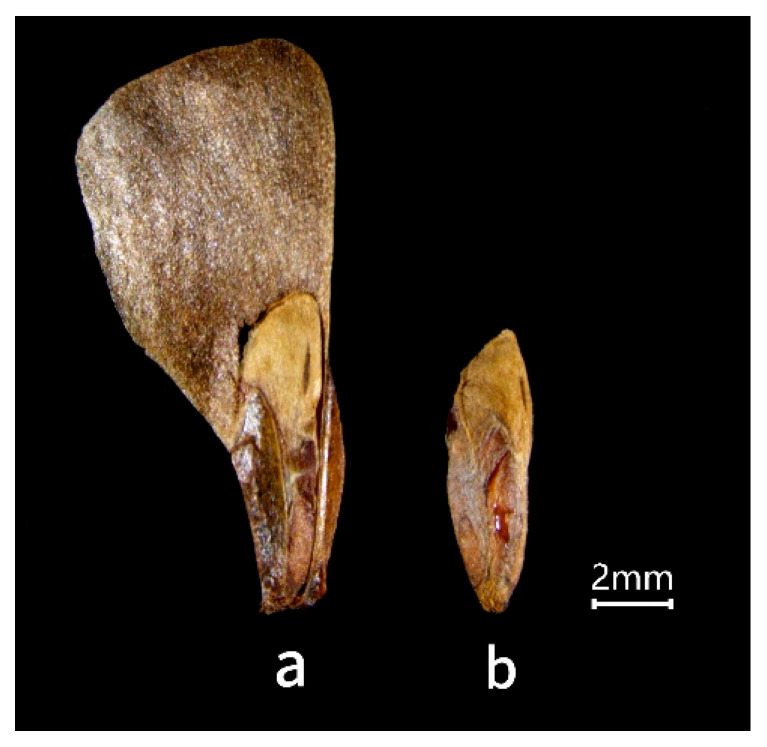
(**a**) Morphology of intact seed and (**b**) de-winged seed of *Abies georgei* var. *smithii*.

Smith fir (*Abies georgei* var. *smithii*) seeds are elongated ovoids with translucent membranous wings, the base not fully covered by the wings (Figure 1). Intact seeds averaged 11.52 ± 0.01 mm in length and 7.23 ± 0.03 mm in width, while de-winged seeds had a mean length of 3.01 ± 0.03 mm. The thousand-seed weight was 8.59 ± 0.03 g.

### 2.2. Effect of Temperature and Light on Seed Germination

**Figure 2:**
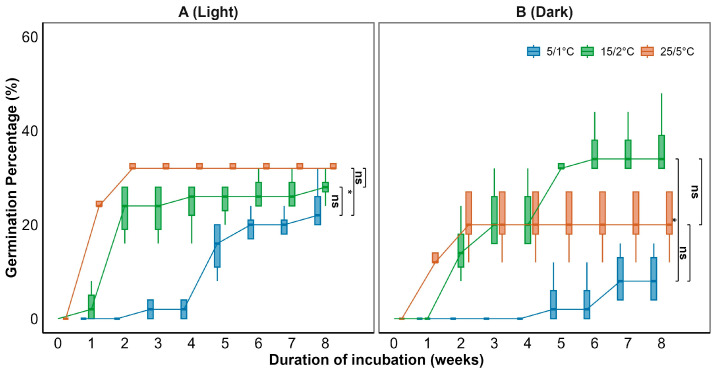
Germination percentage of intact *Abies georgei* var. *smithii* seeds incubated for 8 weeks at various temperature regimes in (**A**) light (12 h photoperiod) and (**B**) continuous darkness. Data in figures are mean ± s.e. (n = 4). Differences among temperature treatments were tested using one-way ANOVA with Tukey’s HSD (* *p* < 0.05; ns, not significant).

**Figure 3:**
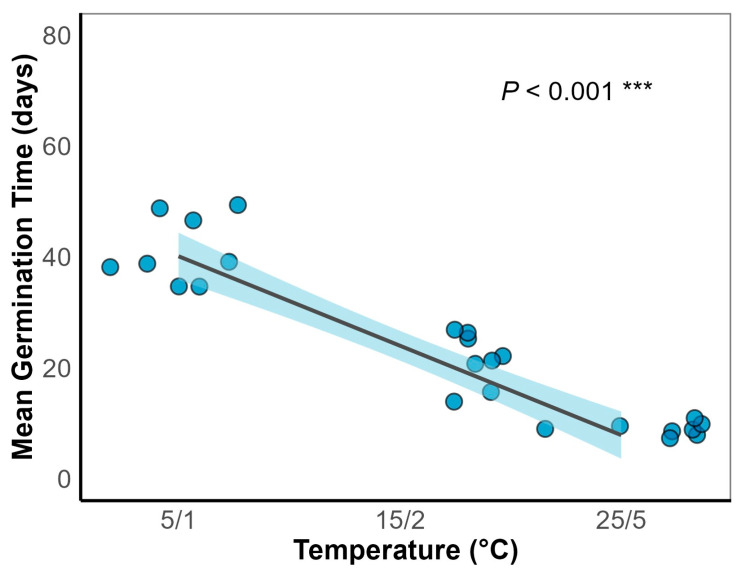
Effect of temperature regimes (5/1, 15/2, and 25/5 °C) on mean germination time (days) of *Abies georgei* var. *smithii* after incubation for 8 weeks; *** indicates a statistically significant relationship at *p* < 0.001 (Generalized linear model).

Generalized linear models (GLMs) showed that temperature significantly affected final germination percentage (*p* < 0.001; Figure 2 and Table 1). Temperature also significantly influenced mean germination time (MGT) (*p* < 0.001; Figure 3). Germination increased with temperature, reaching a maximum of 37.0 ± 3.8% at 15/2 °C (Figure 2B). MGT decreased with temperature consistently under both light and dark conditions, indicating that higher temperatures accelerated germination (Figure 3). A significant interaction between temperature and light was observed (*p* < 0.05; Table 1). Light significantly enhanced germination at lower temperatures (Figure 2A), reaching 24.0 ± 2.8% under the 5/1 °C regime in light, compared to only 9.0 ± 3.0% under darkness. This suggests that light positively regulated germination under cold conditions.

### 2.3. Effect of Seed Wings on Seed Germination

**Figure 4:**
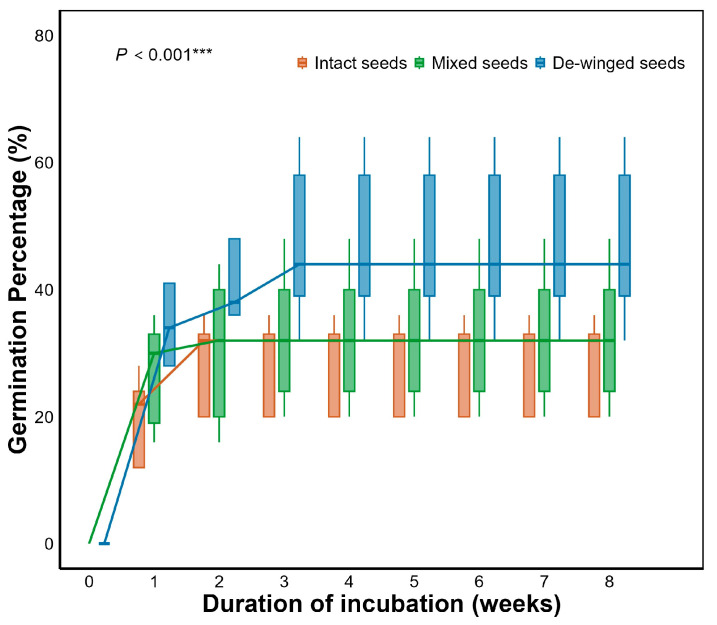
Germination percentages of intact seeds, mixed and de-winged seeds for *Abies georgei* var. *smithii*. Intact, mixed and de-winged seeds denote untreated seeds, de-winged seeds mixed with their detached wings, and seeds with wings removed, respectively. Data in figures are mean ± s.e. (n = 4). *** significant effect of treatment at *p* < 0.001 (Generalized linear model).

**Figure 5:**
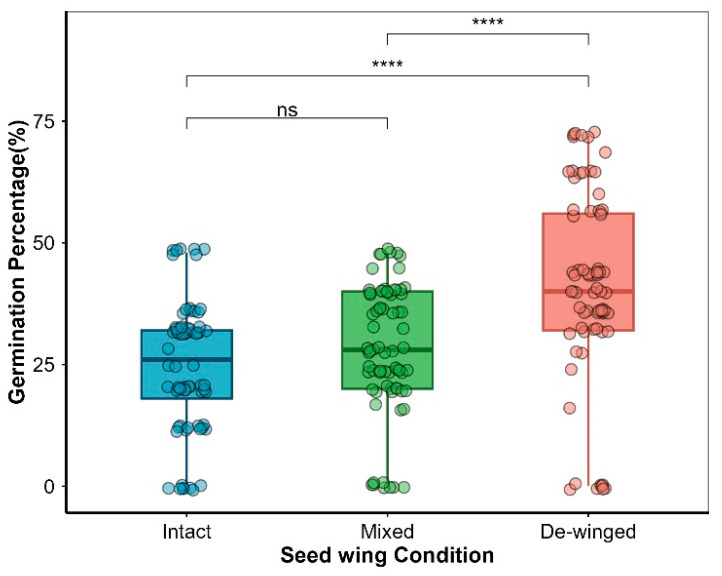
Results of Paired *t*-tests to test for differences in germination percentages of *Abies georgei* var. *smithii* seeds under different seed wing treatments. Intact seeds refer to seeds in their original state. Mixed seeds refer to de-winged seeds mixed with their detached wings. De-winged seeds refer to seeds with their wings removed. ns and **** indicate non-significant and significant results at *p* < 0.0001 among different seed wing treatments, respectively.

GLM results showed that seed wings significantly affected seed germination in Smith fir (*p* < 0.001, Table 1). The germination percentage of de-winged seeds reached 48.5 ± 5.0%, significantly higher than that of intact seeds (26.0 ± 2.4%) (*p* < 0.001; Figure 4 and Figure 5), suggesting that seed wings inhibit germination. No significant difference was found between mixed and intact seeds, but the germination percentage of de-winged seeds was significantly higher than mixed seeds (32.5 ± 3.5%) (*p* < 0.001; Figure 5).

## 3. Discussion

This study addresses the key questions regarding the dormancy and seed wing-mediated regulation of Smith fir (*Abies georgei* var. *smithii*) germination. We found that Smith fir seeds exhibit conditional dormancy, with germination delayed under cold temperatures and facilitating synchronization with spring snowmelt, as hypothesized. Temperature and light were identified as critical environmental cues: higher temperatures accelerated germination, while light promoted germination under cooler conditions, aligning seedling emergence with favorable spring conditions [3]. In addition to these environmental factors, seed wings play a significant role in inhibiting germination, likely through the release of chemical inhibitors. This dual mechanism of environmental regulation and wing-mediated inhibition works together to synchronize seedling emergence with the optimal spring growing season, ensuring successful establishment in harsh subalpine habitats.

### 3.1. Seed Morphological Characteristics and Their Ecological Implications

**Figure 6:**
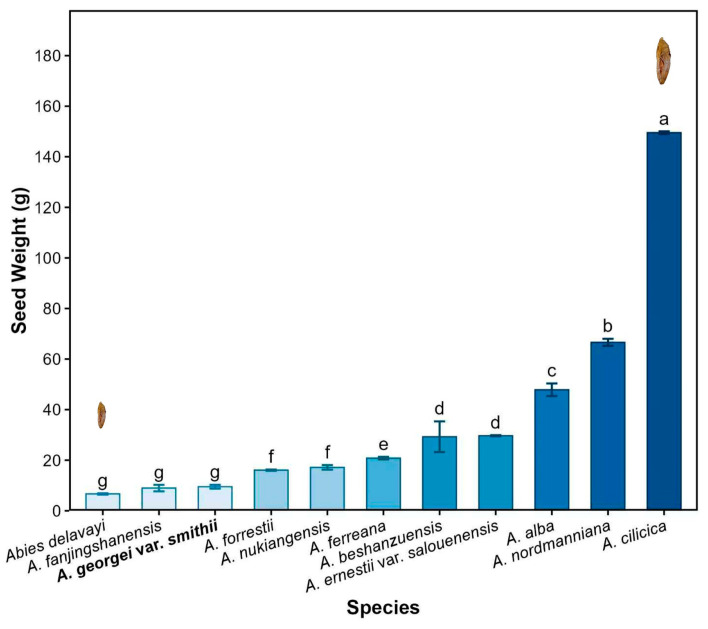
Variation in seed weight among *Abies* species. Data in figures are mean ± s.e. Lowercase letters (a–g) indicate significant differences among species (*p* < 0.05, Tukey’s HSD test). Bar color intensity increases with seed weight. Seed weight refers to the thousand-seed weight.

**Figure 7:**
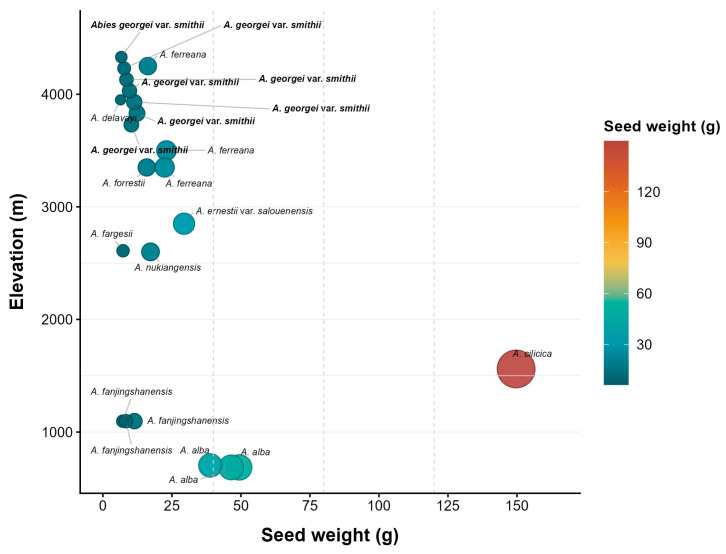
Relationship between seed weight and elevation in *Abies* species. Bubble size and color represent seed weight. Seed weight refers to the thousand-seed weight.

Smith fir seeds are elongated ovoids with translucent membranous wings, a typical feature among conifers that aids in wind dispersal. In this study, the thousand-seed weight of Smith fir was 8.59 ± 0.03 g, which lies toward the lower end of the reported range for *Abies* species (6–149 g) (Figure 6) [21,22,23]. Typically, higher elevations tend to produce smaller seeds [24], a pattern also observed in *Abies* species (Spearman’s ρ = −0.507, *p* = 0.016) (Figure 7). Although reduced seed mass limits nutrient reserves, it likely confers an ecological advantage by enhancing wind dispersal efficiency. This pattern reflects a trade-off between dispersal efficiency and offspring provisioning, suggesting that lighter seeds may facilitate greater colonization potential under alpine treeline conditions [25].

The small seed size and large wings of Smith fir strike a balance between stress resistance and dispersal efficiency. Larger seeds generally provide better seedling establishment in harsh conditions, but the smaller seeds of Smith fir with large wings are more effectively dispersed by wind, facilitating colonization in high-altitude habitats. This trade-off in seed characteristics supports its survival and establishment at altitudes above 4400 m [26], making Smith fir one of the highest-elevation forest species globally [27,28].

### 3.2. Response of Seed Germination to Temperature and Light

Temperature is a critical environmental factor regulating seed germination [29]. In this study, Smith fir seeds exhibited optimal germination (37.0 ± 3.8%) at 15/2–25/5 °C, with increasing temperatures significantly enhancing germination percentages and reducing mean germination time (MGT) (Figure 2 and Figure 3), indicating strong thermal adaptability. The initial germination temperature requirement being relatively high in some alpine plants is explained in terms of adaptation to environmental cues that ensure seeds germinate only when conditions are favorable for seedling survival [30,31].

Light significantly affected germination under low-temperature conditions (*p* < 0.05). Light promoted germination under low temperatures (5/1 °C), whereas its effect diminished at higher temperatures, where germination remained high regardless of light (Figure 2). This pattern suggests a seasonal shift in germination cues, from light-mediated initiation in early spring to temperature-driven germination later in the season. This indicates that light acts as an important cue under suboptimal temperature conditions, signaling the onset of favorable conditions after snowmelt. Similar light–temperature interactions have been observed in other temperate and alpine species, such as *Corylus avellana*, where light promotes germination during early spring when soils are cold but snow-free [32,33]. Such a light-mediated response likely enables Smith fir seeds to germinate soon after snowmelt, maximizing the limited window for seedling establishment within the short subalpine growing season.

### 3.3. Seed Dormancy Type

According to the dormancy classification by Baskin & Baskin [3], Smith fir appears to exhibit conditional dormancy (CD). Seeds germinated readily at 15/2 °C and 25/5 °C, but were substantially delayed under 5/1 °C. However, after four weeks at 5/1 °C, germination percentages significantly increased, particularly under light. This suggests that prolonged cold exposure acts as a stratification cue for dormancy release. Cold-induced dormancy release has been reported in many other alpine species, including *Primula* [34] and *Jeffersonia dubia* [35]. These findings highlight an adaptive germination strategy of Smith fir that enables seedling establishment under the harsh conditions of high-altitude subalpine environments.

### 3.4. Ecological Role of Seed Wings in Germination

Seed appendages are widely recognized to influence germination dynamics across plant lineages, yet their ecological function in gymnosperms remains comparatively understudied. Our results demonstrate that seed wings significantly inhibited final germination of Smith fir seeds (*p* < 0.001), with the peak germination percentage increasing from 48% in intact seeds to 72% following wing removal (Figure 4). This substantial increase indicates that the seed wing acts as a regulatory structure delaying germination. Similar inhibitory effects of persistent appendages have been reported in several angiosperms, including *Atriplex*, *Salsola*, *Acer*, and *Haloxylon*, where removal of bracteoles or perianths enhanced germination percentages [14,36,37,38], suggesting a functional similarity of appendages in fine-tuning germination timing and soil seed bank persistence. Notably, complex dormancy mechanisms and low germination percentages are pervasive constraints on natural regeneration across the genus *Abies* [39,40,41,42]. Research across diverse *Abies* species—ranging from the endangered *A. nebrodensis* [40] and *A. marocana* [41] to *A. hickelii* [39] and the widespread *A. balsamea* [42]—reveals that the characteristically low germination percentages in this genus are not due to low seed viability, but rather reflect a strategy of strictly regulated germination timing to ensure regeneration success. Our observation of wing-mediated chemical inhibition in Smith fir offers a plausible mechanistic explanation for this genus-wide phenomenon, suggesting that such chemical regulation may represent a phylogenetically conserved trait within the genus. On a broader evolutionary scale, a similar regulatory function is also observed in the gymnosperm *Welwitschia mirabilis*, where dry bracts constrain germination under suboptimal conditions, functioning analogously to the inhibitory perianths of certain angiosperms [43]. Taken together, these findings indicate that regulation of germination via appendages is not a taxon-specific trait but rather a convergent evolutionary strategy reflecting a conservative life–history trade-off between “spatial dispersal” and “temporal establishment” [44,45]. Comparable trade-offs have also been documented in Pinaceae cones, where mechanical and hydraulic properties of the cone scales determine seed release timing and dispersal efficiency [46].

Interestingly, the mixed treatment revealed no significant difference in germination relative to intact seeds, suggesting that the inhibitory effect is not primarily physical (e.g., mechanical restraint or reduced water uptake) [47]. Instead, the inhibition is plausibly mediated by water-soluble endogenous compounds leaching from the wings, analogous to the chemical inhibition reported in the bractlets of *Atriplex griffithii* [38]. Future chemical analyses of *Abies* wing exudates are warranted to confirm the presence and identity of such inhibitory substances, which may include phenolics or resin derivatives known from *Abies* seed coats [48].

The ecological function of seed wings appears to be context-dependent and shaped by environmental filtering. In xeric habitats, structures such as pappi or pubescence promote rapid water uptake, facilitating germination following ephemeral rainfall [49,50]. In contrast, for subalpine species such as Smith fir, thick and resinous wings act as biochemical barriers delaying germination under transiently favorable autumn temperatures. In its natural high-elevation habitat, these wings likely prevent premature germination during short warm spells, while conditional dormancy further inhibits emergence during winter. As snowmelt occurs in spring, the wings gradually soften, decompose, and leach inhibitory compounds, synchronizing germination with optimal moisture and nutrient availability. Such synchronization mirrors the adaptive strategies seen in other montane *Pinaceae* species, where dispersal mode and phylogeny jointly constrain germination timing [51]. The trade-off between dispersal and establishment reflects a central theme in conifer ecology: structures that enhance dispersal (e.g., wings, lightweight cones) may simultaneously delay germination to ensure seedling survival in unpredictable alpine climates. Similarly, recent studies on *Juniperus deppeana* (Cupressaceae) suggest that seed coverings may chemically or mechanically inhibit germination until specific cues—such as passage through animal guts—are encountered [52].

### 3.5. Integrated Control, Mismatches, and Future Perspectives

The germination of Smith fir appears to be governed by a sequential gating system integrating morphological, chemical, and physiological layers. In this model, the seed wing functions as the primary biochemical barrier during autumn, preventing premature germination. Prolonged winter chilling alleviates physiological dormancy, while spring snowmelt provides the dual cues of moisture and inhibitor leaching. This synchronization ensures that seedling emergence coincides with the short alpine growing season, when soil nutrients and water availability peak [3,51]. However, such a finely tuned system may be increasingly vulnerable to climatic shifts. Earlier snowmelt or erratic spring precipitation—both linked to ongoing warming—could lead to premature inhibitor leaching and expose germinants to late frost events. These phenological mismatches threaten to reduce recruitment success and shift population boundaries at the treeline [53]. Given these complexities, we consider that elucidating the ecological patterns of germination strategies is a critical prerequisite before delving into biochemical mechanisms. This study serves as an essential cornerstone, establishing the necessary morphological and ecological framework to guide future targeted physiological research. Building on this, future research combining field experiments with physiological and biochemical analyses is essential to identify these inhibitors and forecast how Smith fir and related conifers adjust regeneration strategies under climate change.

## 4. Materials and Methods

### 4.1. Study Area

Seed collection was conducted in the Sygera Mountains, Nyingchi Prefecture, Tibet Autonomous Region (29°10′ N–30°15′ N, 93°12′ E–95°35′ E), at elevations ranging from 3200 to 4728 m. The region is characterized by a subalpine, cold-temperate, semi-humid climate and represents the typical habitat of Smith fir.

### 4.2. Seed Collection

Seeds were collected in late October 2023 from 30 reproductively mature, healthy Smith fir trees, randomly selected within a natural forest stand. To minimize genetic similarity, trees were spaced at least 50 m apart. Mature cones were harvested using a pole pruner from sun-exposed branches to ensure the collection of physiologically mature seeds.

### 4.3. Seed Extraction

Cones were air-dried at room temperature (ca. 15 °C) for 10 days to promote natural seed dehiscence. Once open, seeds were manually extracted by gently shaking and tapping the cones. Debris, cone scales, and damaged seeds were removed by hand. Approximately 5000 seeds were obtained and stored under cool, dry conditions until germination experiments in November 2023.

### 4.4. Seed Morphological Characteristics

The length and width of intact and de-winged seeds were measured using a vernier caliper (0.01 mm precision). Fifty seeds per treatment were measured for three replicates, and the results were averaged. Seed weight was determined as the mass of 1000 seeds using an analytical balance (0.0001 g precision). One thousand seeds per replicate were randomly selected, and the measurement was repeated three times to obtain the average weight. To investigate interspecific variations, data on seed weight and elevation for other *Abies* species were compiled from published studies [21,22,23].

### 4.5. Germination Experiments Under Different Temperature and Light Conditions

To assess the impact of temperature and light on seed germination, seeds were incubated under three alternating temperature regimes: 5/1 °C (representing October and May), 15/2 °C (June and September), and 25/5 °C (July to August), reflecting the daily mean maximum and minimum temperatures during the growing season at the study site [54]. For each temperature regime, two light conditions were tested: a 12 h light/12 h dark photoperiod (simulating spring to autumn conditions), and continuous darkness (simulating low-light environments such as snow or forest litter). Petri dishes for the dark treatment were wrapped in aluminum foil to block light. Seeds were placed in 60 mm Petri dishes lined with two layers of filter paper moistened with distilled water. Each treatment had four replicates of 25 seeds. Germination, defined as radicle emergence (≥2 mm), was monitored weekly for a period of eight weeks. Observations for the dark treatment were conducted under a dim green safe light.

### 4.6. Effect of Seed Wings on Germination

To evaluate the effect of the seed wings, three different treatments were applied: intact seeds, mixed seeds (wings removed and mixed with the seeds), and de-winged seeds. Seeds were placed in 60 mm Petri dishes with two layers of filter paper moistened with distilled water, and incubated under an alternating temperature regime of 25/5 °C (12 h light/12 h dark). Each treatment included four replicates of 25 seeds, and germination was monitored weekly as described above.

Mean germination time (MGT) was calculated using the formula from Ellis & Roberts [55]:

(1) $$\mathrm{MGT}\;=\;\frac{\sum Dn}{\sum n}$$

where *n* is the number of seeds that germinate on the *D*-th day, and *D* is the number of days counted from the beginning of the experiment.

### 4.7. Statistical Analyses

Statistical analyses were performed in R (4.3.0). Data preprocessing and organization were performed using the tidyr [56] and dplyr packages [57], and visualized using ggplot2 [58]. Generalized linear models (GLMs) were fitted using the stats package [59] to evaluate the effects of light, temperature, and seed wings on seed germination percentages (binomial error distribution, logit link function) and mean germination time (Gaussian error distribution, log link function). The significance of main effects, interactions, and differences among seed wing treatments were assessed using Chi-square tests and paired *t*-tests implemented in the stats package. Germination percentages among temperature treatments and seed weight differences among *Abies* species were analyzed using one-way ANOVA with Tukey’s HSD test, utilizing the rstatix [60] and agricolae [61] packages, respectively. The relationship between seed weight and elevation was evaluated using Spearman’s rank correlation analysis.

## 5. Conclusions

This study elucidates the mechanisms by which Smith fir (*Abies georgei* var. *smithii*) seeds regulate germination timing through interactions between environmental cues and intrinsic traits. Germination is governed by conditional physiological dormancy that is released by exposure to low temperatures and influenced by light availability, while wing-associated chemical inhibitors impose additional constraints. This suggests an evolutionary trade-off between wind dispersal efficiency and temporal regulation of seedling establishment. The integration of these physiological and morphological mechanisms optimizes germination timing to coincide with favorable spring conditions, thereby enhancing seedling establishment in high-altitude subalpine forests. Collectively, these findings highlight how dormancy regulation and seed morphology interact to shape regeneration strategies of conifers in cold mountain environments.

## Figures and Tables

**Table 1:** Effect of seed wings, light, temperature and the light × temperature interaction on seed germination of *Abies georgei* var. *smithii*.

Variables	*χ* ^2^	df	*p*
Seed wings	33.73	2	<0.001 ***
Light	0.48	1	0.489
Temperature	15.43	2	<0.001 ***
Temperature × Light	9.91	2	<0.05 **

**Note:** Results from a GLM (Generalized linear model) with binomial distribution. *χ*^2^ values are from likelihood ratio tests. ** *p* < 0.05, *** *p* < 0.001.

## Data Availability

The data presented in this study are available on request from the corresponding author. The data are not publicly available due to planned future analyses.
